# Chloroform in Puerperal Convulsions

**Published:** 1853-08

**Authors:** 


*Chloroform in Puerperal Convulsions.*—Dr. Van Buren reported to the New York medical and Surgical Society, a case of puerperal convulsions, occurring in a lady, twenty-five years of age, with her first child. During gestation, she had enjoyed her ordinary health, except on the evening preceding the attack, when she had retired with a headache. Her physician was sent for at half past one o'clock, A. M., and found her in a convulsion.

Labor had not commenced. An hour later Dr. Van B. was called, and found her in her third convulsion. On examination per vaginam, it was ascertained that the os uteri was beginning to dilate. Chloroform was immediately given and after a time the convulsions were relieved, when it was "let up," and the convulsions again returned. Its use was again resumed, and she was kept under its influence until about six o'clock, A. M., at which time the os was found to be sufficiently dilated to admit of interference. The forceps was applied, and the delivery speedily completed. The patient was then left in a comfortable condition. Another convulsion, however, came on shortly afterward, and they recurred every twenty minutes, except when chloroform was given, which controlled them. Other appropriate remedies were resorted to, but the anæsthetic was mainly relied upon. Its use was continued until a few hours before death, which occurred fifty-two hours after the first seizure. During all this time there was no secretion of urine. The child is alive and doing well.

Dr. Metcalf asked if the coming on of headache with the commencement of labor, where the patient had up to that time enjoyed good health, was not of grave import? He then mentioned a case in which convulsions followed this symptom.

Dr. Delafield replied that he should be alarmed, and adopt precautionary measures; and alluded to a case of this kind which had fallen under his observation during the past week. In this connection, he inquired of the Society, the result of their experience in the use of chloroform in puerperal convulsions.

Dr. Metcalf replied, that in a half dozen cases, occurring in his own practice, and another half dozen in the practice of his friends, when chloroform had been relied upon, but one case had terminated fatally.

Dr. McCready has seen one case in which it was administered freely, but did not control the convulsions. This case, however, terminated favorably.

Dr. Delafield narrated a case in which it was given without at all controlling the disease, and the result was fatal. He doubts the advantage of chloroform in this disease; thinks that, under proper treatment, the great majority of cases of puerperal convulsions will terminate favorably. He recommends one free bleeding from the arm at the commencement of the attack, followed by cupping and strong counter-irritation about the head itself. In the only case which terminated fatally in his practice last year, chloroform was used.

Dr. Metcalf thinks that the tendency to a recurreuce of the convulsions is owing to excessive nervous excitability; and as chloroform will undoubtedly allay this condition, he thinks its use highly beneficial. He then mentioned a case of his own, in which the patient had but one convulsion, which terminated fatally. A *post-mortem* examination revealed a clot in the brain. This he considers rare.

Dr. Delafield, in reply to questions put by several members, stated that he does not consider opium as a safe remedy in this disease. A very large proportion of cases occur after the labor is completed; and they very seldom occur before the labor has fairly commenced. Convulsions, however, sometimes do occur, from time to time, during gestation, and yet the labor goes on perfectly well.

Dr. Markoe inquired what influence the progress of labor produces upon the convulsions. Also as to the propriety of hastening the labor in such cases.

Dr. Delafield replied that he would never interfere when there would be any serious difficulty in doing so—where there would be much shock; but would wait until the os uteri was well dilated. He also remarked that children are not often born living after convulsions have existed for any length of time. Where convulsions occur and subside before delivery, the child is always dead.—*New York Medical Times*, *April*, 1853.

